# Admixture Fine-Mapping in African Americans Implicates *XAF1* as a Possible Sarcoidosis Risk Gene

**DOI:** 10.1371/journal.pone.0092646

**Published:** 2014-03-24

**Authors:** Albert M. Levin, Michael C. Iannuzzi, Courtney G. Montgomery, Sheri Trudeau, Indrani Datta, Indra Adrianto, Dhananjay A. Chitale, Paul McKeigue, Benjamin A. Rybicki

**Affiliations:** 1 Department of Public Health Sciences, Henry Ford Health System, Detroit, Michigan, United States of America; 2 Department of Medicine, Upstate Medical University, Syracuse, New York, United States of America; 3 Arthritis and Clinical Immunology Research Program, Oklahoma Medical Research Foundation, Oklahoma City, Oklahoma, United States of America; 4 Department of Pathology, Henry Ford Health System, Detroit, Michigan, United States of America; 5 Public Health Sciences Section, University of Edinburgh Medical School, Edinburgh, Scotland, United Kingdom; National Cancer Institute, National Institutes of Health, United States of America

## Abstract

Sarcoidosis is a complex, multi-organ granulomatous disease with a likely genetic component. West African ancestry confers a higher risk for sarcoidosis than European ancestry. Admixture mapping provides the most direct method to locate genes that underlie such ethnic variation in disease risk. We sought to identify genetic risk variants within four previously-identified ancestry-associated regions—6p24.3–p12.1, 17p13.3–13.1, 2p13.3–q12.1, and 6q23.3–q25.2—in a sample of 2,727 African Americans. We used logistic regression fit by generalized estimating equations and the MIX score statistic to determine which variants within ancestry-associated regions were associated with risk and responsible for the admixture signal. Fine mapping was performed by imputation, based on a previous genome-wide association study; significant variants were validated by direct genotyping. Within the 6p24.3–p12.1 locus, the most significant ancestry-adjusted SNP was rs74318745 (p = 9.4*10^−11^), an intronic SNP within the *HLA-DRA* gene that did not solely explain the admixture signal, indicating the presence of more than a single risk variant within this well-established sarcoidosis risk region. The locus on chromosome 17p13.3–13.1 revealed a novel sarcoidosis risk SNP, rs6502976 (p = 9.5*10^−6^), within intron 5 of the gene X-linked Inhibitor of Apoptosis Associated Factor 1 (*XAF1*) that accounted for the majority of the admixture linkage signal. Immunohistochemical expression studies demonstrated lack of expression of XAF1 and a corresponding high level of expression of its downstream target, X-linked Inhibitor of Apoptosis (*XIAP*) in sarcoidosis granulomas. In conclusion, ancestry and association fine mapping revealed a novel sarcoidosis susceptibility gene, *XAF1*, which has not been identified by previous genome-wide association studies. Based on the known biology of the XIAP/XAF1 apoptosis pathway and the differential expression patterns of XAF1 and XIAP in sarcoidosis granulomas, we suggest that this pathway may play a role in the maintenance of sarcoidosis granulomas.

## Introduction

Sarcoidosis is a granulomatous, inflammatory disease of uncertain etiology. The lung is the most commonly affected organ, with 90% of cases presenting pulmonary involvement [1]. The development and accumulation of granulomas—compact, centrally-organized collections of macrophages and epithelioid cells encircled by lymphocytes—constitute the fundamental abnormality in sarcoidosis. Despite the lack of a known etiologic agent, epidemiologic and molecular studies indicate that sarcoidosis is an antigen-driven disease[2], with a Th1- and possibly Th17-mediated immune response[3]. Although patients with lung involvement may not progress sequentially through the Scadding disease stages (I–IV) [4], pulmonary sarcoidosis often begins as asymptomatic bihilar lymphadenopathy (Stage I) and may progress to overt pulmonary involvement, as seen in Stages II and III. Stage IV sarcoidosis is characterized by pulmonary fibrosis and lack of immune cell activity; although death from sarcoidosis is rare, Stage IV cases have lower rates of survival [5].

Populations of West African descent have higher sarcoidosis incidence than European populations; the adjusted annual incidence among African Americans is roughly three times that of White Americans (35.5/100,000 versus 10.9/100,000) [6]. African ancestry is also associated with more chronic and severe disease [7], [8]. In recently admixed populations (such as African Americans), mapping by admixture linkage disequilibrium takes advantage of such differences in disease susceptibility between ancestral populations to identify genetic loci associated with both disease and ancestry [9], [10]. Current admixture mapping methods permit estimation of local ancestry (defined as zero, one, or two copies of a given ancestral origin) over a dense set of genetic markers [11]. In addition to refining an ancestry signal, these methods of local ancestry estimation also permit testing whether variation at a single SNP accounts for a local ancestry signal [11]. Compared to the genome-wide association approach, association testing within regions of admixture linkage improves statistical power by greatly limiting the number of tests performed and allows for discovery of monomorphic variants in parent populations.

In our previous admixture mapping scan for sarcoidosis risk loci in African Americans, we identified nine regions that suggested admixture linkage to both West African as well as European alleles [12]. Upon further analysis that included additional related subjects, four of these nine regions increased in statistical significance, while the remaining five regions decreased in significance [13]. The strongest admixture signal was located at chromosome 6p24.3–12.1, the locus encompassing the human leukocyte antigen (*HLA*) region, which is known to be associated with sarcoidosis risk [14]. The most significant novel risk locus was found at chromosome 17p13.3–13.1 [12]. Both loci showed an association between increased African ancestry and sarcoidosis risk. Three additional regions (2p12–q12.3, 10p12.2–10q11.23, and 16q22.1–16q23.2) showed suggestive heterogeneity in ancestry linkage between cases whose disease resolved within two years of diagnosis compared to those with fibrotic lung disease (Stage IV). In our original genome-wide association study (GWAS), genome-wide significant effects were confined to *HLA* region [14]. The goal of the present study was to leverage the independent local ancestry information used by admixture mapping to identify specific SNP(s) most likely to account for the observed ancestry signal within the *HLA* region. In addition, we sought to fine map novel regions missed in the GWAS to guide gene sequencing and/or functional studies of additional putative risk genes and genes associated with lung fibrosis, which was not a component of the original GWAS. To quantify the contribution of local West African ancestry to sarcoidosis risk, we used the genome-wide complex trait analysis (GCTA) approach to estimate the heritability of sarcoidosis due to local ancestry across autosomes [15], [16]. To achieve these goals, we used local ancestry and genotype imputation based on data from our previous African American GWAS of sarcoidosis risk.

## Results

### Fine mapping results for sarcoidosis risk and Scadding stage IV regions

Table 1 displays results for markers within regions of sarcoidosis ancestry risk linkage that displayed the most significant allelic association, before and after adjustment for local ancestry. *(A complete list of association results with local ancestry-adjusted or -unadjusted marker p-values<0.05 is displayed in Table S1.)* Three of the four admixture linkage regions (6p24.3–12.1, 17p13.3–13.1, and 2p12–q12.1) contained variants that were associated with sarcoidosis risk at or below the suggestive level of genome-wide significance (p = 10^−5^).

**Table 1:** Peak allelic associations within genomic regions of sarcoidosis ancestry linkage after adjustment for both global and local West African ancestry and corresponding MIX score results.

						Global Ancestry Adjusted			Global + Local Ancestry Adjusted			MIX
SNP,Allele^1^,Status^2^	Locus	f_CEU_	f_AFR_	f_AFF_	f_UNF_	OR	95%CI	P	OR	95%CI	P	P
rs62158012, A/C, imputed	Chr 2q11.2, MGAT4A intron	0.27	0.01	0.09	0.06	1.70	1.38–2.09	6.7*10^−7^	1.70	1.36–2.12	2.8*10^−6^	2.5*10^−5^
rs74318745, A/G, genotyped	Chr 6p21.32, HLA-DRA intron	0.51	0.42	0.42	0.51	0.69	0.62–0.78	9.4*10^−11^	0.69	0.62–0.77	4.5*10^−11^	7.9*10^−12^
rs78512816, C/T, genotyped	Chr 6q23.3, 22 kb downstream of OLIG3	---3	0.07	0.03	0.05	0.58	0.44–0.76	9.5*10^−5^	0.59	0.45–0.78	1.8*10^−4^	9.8*10^−5^
rs6502976, C/G, imputed	Chr 17p13.1, XAF1 intron	0.65	0.09	0.18	0.23	0.74	0.64–0.84	9.5*10^−6^	0.74	0.63–0.86	1.2*10^−4^	7.9*10^−5^

Abbreviations: f_CEU_: frequency of modeled allele in HapMap Northern and Western European ancestry population; f_AFR_: frequency of modeled allele in HapMap Yoruban African ancestry population; f_AFF_: frequency of modeled allele in sarcoidosis-affected individuals; f_UNF_: frequency of modeled allele in unaffected individuals; OR: odds ratio; 95%CI: 95% confidence interval; P: p-value; MIX: MIXSCORE test.

1 Minor allele in African Americans is bolded; modeled by generalized estimating equations adjusting for percent global West African ancestry and sex.

2 “Imputed” Indicates a SNP that was imputed rather than directly genotyped. Accuracy of imputation was assessed for SNPs with p-values<10^−5^ in a sub-sample, and for each SNP; agreements overall and by genotype are reported in Table S2. Overall accuracy of imputation was 98.7% (rs62158012) and 98.0% (rs6502976).

3 No carriers of the T allele of rs78512816 exist within HapMap and 1000 Genomes Project European populations.

**Figure 1:**
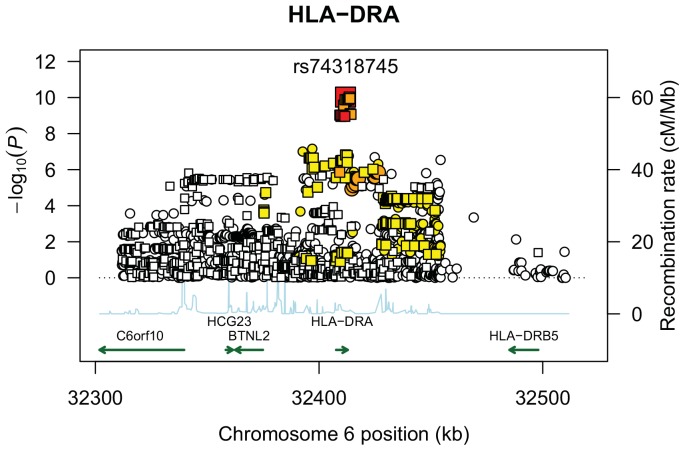
Plot of association test results across chromosome 6p12.1–24.3. The –log_10_ (P-values) plotted are from SNP association tests adjusted for global percent African ancestry and sex. Association p-values plotted with squares indicate genotyped SNPs; circles indicate imputed SNPs. Shading indicates linkage disequilibrium (LD) r^2^ values between SNP rs74318745 and the remaining SNPs in the region (strong LD: r^2^≥0.8 (red); moderate LD: r^2^≥0.5 (orange); weak LD: 0.8>r^2^>0.5 (yellow); not in LD: r^2^<0.2 (white)) were estimated in a sample of 250 unrelated African American controls from the current study. Recombination rates are displayed in blue and are based on the average across the phase II International HapMap reference populations.

The most significant SNPs within 6p24.3–12.1 were located within or near the *HLA-DRA* gene (Figure 1). Of these, the most significant was rs74318745, located within intron 4 of *HLA-DRA* (OR = 0.69; 95% Confidence Interval (CI) 0.62–0.78; p = 9.4*10^−11^). Adjustment for local ancestry showed no confounding (OR = 0.69; CI 0.62–0.77; p = 4.5*10^−11^). Consistent with this finding, the MIX score result for this SNP (p = 7.9*10^−12^) was the most significant in the region, indicating that it is the variant most likely to explain the admixture linkage signal. Further, the DIFF score p-value (0.051) suggests that one or more additional SNPs in this region contribute to the admixture linkage, as this p-value falls slightly above the nominal significance threshold of 0.05.

SNP rs7431874 is in perfect linkage disequilibrium (r^2^ = 1) with the SNP rs2227139, the most significant SNP identified within the *HLA* region in our GWAS [14]. In that study, subsequent conditional analyses revealed four additional independent variants (SNPs rs146146117 *HLA-DQA1*, rs9461776 *HLA-DRB1*, rs715299 *NOTCH4*, and rs9272320 *HLA-DQA1*) associated with sarcoidosis risk in the *HLA* class II region at the suggestive GWA significance threshold. In the current study, all five variants had DIFF score p-values<0.06, suggesting that none of the variants alone explain the admixture linkage signal. Consistent with this finding, the case-control local ancestry association remained significant after adjustment for each SNP (all ancestry association p-values<0.03). However, adjustment for all five SNPs resulted in a non-significant ancestry association (p = 0.25).

**Figure 2:**
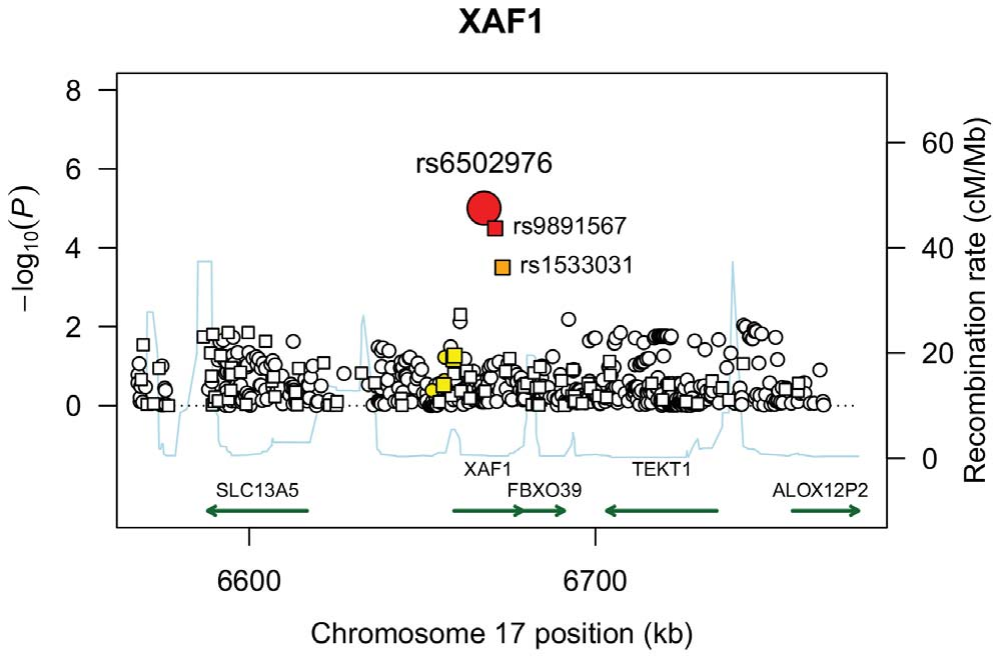
Plot of association test results across chromosome 17p13.1–13.3. The –log_10_ (P-values) plotted are from SNP association tests adjusted for global percent African ancestry and sex. Association p-values plotted with squares indicate genotyped SNPs; circles indicate imputed SNPs. Shading indicates linkage disequilibrium (LD) r^2^ values between SNP rs6502976 and the remaining SNPs in the region (strong LD: r^2^≥0.8 (red); moderate LD: r^2^≥0.5 (orange); weak LD: 0.8>r^2^>0.5 (yellow); not in LD: r^2^<0.2 (white)) were estimated in a sample of 250 unrelated African American controls from the current study. Recombination rates are displayed in blue and are based on the average across the phase II International HapMap reference populations.

The second most significant admixture linkage region was 17p13.3–13.1, with multiple SNPs associated with sarcoidosis risk and no evidence of confounding by local ancestry. The most significant of these was the imputed SNP rs6502976 (OR = 0.74; CI 0.64–0.84; p-value = 9.5*10^−6^), located within intron 5 of the X-linked inhibitor of apoptosis associated factor 1 (*XAF1*) gene. This finding was supported by the directly-genotyped SNP rs9891567 (Figure 2; OR = 0.79; CI 0.67–0.87 p-value = 3.2*10^−6^), which is in linkage disequilibrium (LD; r^2^ = 0.81). Direct genotyping of rs6502976 demonstrated high concordance (98%, Table S2) with the imputed calls. Adjustment for local ancestry had little effect on the odds ratio (OR = 0.74; CI 0.63–0.86; p = 1.2*10^−4^). The MIX score result (p = 7.9*10^−5^) indicated that this variant was likely to explain the admixture linkage; the corresponding DIFF result (p = 1.00) indicated that it was likely the only one explaining the admixture linkage result. Consistent with this finding, odds ratios were similar across strata of individuals with zero (OR = 0.84, CI 0.45–1.54), one (OR = 0.78, CI 0.59–1.02), and two (OR = 0.74, CI 0.61–0.91) African alleles.

Among the three non-*HLA* admixture linkage loci studied, the most significant association both before and after adjustment for local ancestry (Table 1) was identified within the 2p13.3–2q12.1 locus at the imputed SNP rs62158012, located within an intron of the mannosyl (alpha-1,3-)-glycoprotein beta-1,4-N-acetylglucosaminyltransferase, isozyme A (*MGAT4A*) gene. Similar to the other variants in Table 1, the odds ratio for SNP rs62158012 shows no confounding by local ancestry, and the MIX (p = 2.5*10^−5^) and DIFF (p = 0.64) scores suggest that this variant explains the ancestry signal. Among the genotyped SNPs, rs12467276 is in highest pairwise LD (r^2^ = 0.44) with rs62158012 and consistently reflects its association with risk (OR = 1.36; CI 1.16–1.59; p = 1.4*10^−4^). This region overlaps with 2p12–q12.3, the region of admixture linkage to Scadding stage IV disease. Before adjustment for local ancestry, rs62158012 was associated with risk of stage IV disease (OR = 2.05, CI 1.38–3.05, p = 3.9*10^−4^). After adjustment for local ancestry, the odds ratio suggests that an additional marker exists in this region that may explain the admixture linkage to Scadding stage IV disease (OR = 1.80, CI 1.20–2.71, p = 0.005).

Table 2 contains the association results for markers within regions of Scadding stage IV ancestry linkage. The variant most likely to explain the signal in the 2p13.3–2q12.1 region was imputed SNP rs6547087, which is located within a large intergenic region (Table 2). The MIX (p = 2.2*10^−4^) and DIFF (p = 1.00) scores suggest that there are no additional variants likely to explain the admixture linkage in this region. The genotyped SNP rs2091716 was in high pairwise LD (r^2^ = 0.97) with rs6547087; its effect (OR = 2.02; CI = 1.44–−2.83; p = 4.1*10^−5^) was consistent with it. Among the three regions in our original admixture analysis that were linked to radiographic Scadding stage IV disease, the 10p12.1–11.21 region displayed the highest level of significance in both the unrelated and related analyses. Within this region, SNP rs906233 displayed the most significant local ancestry association (unadjusted OR = 1.77; CI 1.38–2.27; p = 7.7*10^−6^; adjusted OR = 1.70; CI 1.32–2.20; p = 4.8*10^−5^). The MIX score result (p = 3.8*10^−5^) is consistent with this, and the corresponding DIFF result (p = 0.141) suggests that there is not strong evidence for additional variants within the region that account for this signal. Like rs6547087 above, this variant is also located in a gene-poor region; rs906233 is located 69kb upstream of the lysozyme-like 2 (*LYZL2*) gene and 109 kb downstream of the mitogen-activated protein kinase 8 (*MAP3K8*) gene. Among the three Scadding stage IV admixture linkage regions, the most statistically significant association was found in the 16q22.1–23.2 locus at the imputed SNP rs12919626 (Table 2). This SNP is an intronic variant within the fatty acid 2-hydroxylase (*FA2H*) gene. Among the genotyped SNPs, rs11554620 is in highest pairwise LD (r^2^ = 0.20) with rs12919626 and consistently reflect its association with Stage IV disease (OR = 1.35; CI 1.04–1.76; p = 0.024). While there was no evidence of confounding by local ancestry at this locus, the DIFF score (p = 0.004) suggests that at least one additional variant associated with risk of Scadding stage IV disease exists in this region.

**Table 2:** Peak allelic associations within genomic regions of sarcoidosis ancestry linkage with Scadding stage IV disease.

						Global Ancestry Adjusted			Global + Local Ancestry Adjusted			MIX
SNP,Allele^1^,Status^2^	Locus	f_CEU_	f_AFR_	f_AFF_	f_UNF_	OR	95%CI	P	OR	95%CI	P	P
rs6547087, C/T, imputed	Chr 2p12, 270 kb upstream from LRRTM4	0.27	0.03	0.14	0.07	2.21	1.58–3.10	4.4*10^−6^	2.14	1.50–3.07	2.7*10^−5^	2.2*10^−4^
rs906233, A/G, genotyped	Chr 10p11.23, 69 kb upstream from LYZL2	0.39	0.10	0.24	0.15	1.77	1.38–2.27	7.7*10^−6^	1.70	1.32–2.20	4.8*10^−5^	3.8*10^−5^
rs12919626, A/G, imputed^2^	Chr 16q23.1, FA2H intron	0.16	0.08	0.18	0.09	2.33	1.71–3.19	9.6*10^−8^	2.33	1.71–3.19	1.0*10^−7^	2.6*10^−6^
rs145044562^3^, A/T, imputed^2^	Chr 16q23.1, WWOX intron	0.01	---4	0.05	0.02	3.27	1.84–5.81	5.1*10^−5^	3.30	1.85–5.89	5.1*10^−5^	3.6*10^−4^
rs1077963^3^, C/T, imputed^2^	Chr 16q23.1, WWOX intron	0.42	0.05	0.17	0.09	2.02	1.42–2.87	9.9*10^−5^	1.87	1.14–3.05	0.013	3.5*10^−4^

Abbreviations: f_CEU_: frequency of modeled allele in HapMap Northern and Western European ancestry population; f_AFR_: frequency of modeled allele in HapMap Yoruban African ancestry population; f_AFF_: frequency of modeled allele in sarcoidosis-affected individuals; f_UNF_: frequency of modeled allele in unaffected individuals; OR: odds ratio; 95%CI: 95% confidence interval; P: p-value; MIX: MIXSCORE test.

1 Minor allele in African Americans is bolded; modeled by generalized estimating equations adjusting for percent global West African ancestry and sex.

2 Accuracy of imputation was assessed for SNPs with p-values <10^−5^ in a sub-sample; agreements overall and by genotype are reported in Table S2. Overall imputation accuracy was 96.0% (rs6547087), 99.2% (rs906233), and 99.3% (rs12919626).

3 For rs145044562, conditional on rs12919626; for rs1077963, conditional on rs145044562 and rs12919626;

4 No carriers of the A allele of rs145044562 exist within HapMap or 1000 Genomes Project European populations.

To determine whether additional variants could explain the admixture linkage at 16q22.1–23.2 locus, a forward model selection procedure was applied, and the resulting variants are also reported in Table 2. Conditioning on rs12919626, the next most significant SNP in the region is rs145044562 (p = 5.1*10^−5^), which is located within an intron of the WW domain-containing oxidoreductase (*WWOX*) gene. Similar to SNP rs12919626, the DIFF score p-value (p = 0.006) suggests that it is not the only SNP in the region that explains the admixture signal. Further, conditioning on both rs12919626 and rs145044562 revealed a second SNP (rs1077963) within an intron of *WWOX* that was associated with risk of Scadding stage IV disease. The DIFF score for SNP rs1077963 (p = 1.0) suggests that this SNP explains the admixture linkage in this region. Consistent with this finding, the case-control local ancestry association remained significant after adjustment for both rs12919626 and rs145044562 (ancestry association p-values<0.005) but was rendered non-significant (p = 0.62) after adjustment for rs1077963.

### *In silico* expression quantitative trait locus (eQTL) results for *XAF1* SNPs

Because the SNPs most likely to explain the ancestral linkage signals with overall risk and Scadding stage IV disease are found in non-coding or intergenic regions, we used existing eQTL studies to further investigate their possible function. Using the GENe Expression Variation (GeneVar) application [17], we summarized results from two studies of multiple cell types: an eQTL study of 171 female identical twins [18], and a genome-wide study of eQTLs in cord blood samples of 75 individuals [19]. Of the SNPs most likely to explain local ancestry signals, only the SNPs in *XAF1* showed evidence of being cis-acting eQTLs. Results (Table 3) show suggestive evidence for SNP rs6502976 as an eQTL for *XAF1* through linkage disequilibrium with two other SNPs (rs9891567 and rs1533031) that have been directly genotyped in studies of European individuals; both of these SNPs are also associated with risk of sarcoidosis (Figure 2). The pattern of association between these SNPs and XAF1 expression is consistent, with the protective allele at each SNP associated with decreased expression of XAF1. Figures S1 and S2 show XAF1 expression levels by genotype at SNPs rs1533031 and rs9891567, respectively. These findings are also supported by another recent study [20], where rs9891567 was the most significant cis-eQTL for XAF1 transcriptional expression in both B-cells (p = 4.4*10^−20^) and monocytes (p = 1.1*10^−12^).

**Table 3:** Genevar results suggest SNP rs6502976* is an eQTL for *XAF1*.

			Global Ancestry Adjusted					XAF1^3^	
SNP	(r^2^) 1	Alleles^2^	OR	95%CI	P	eQTL Study	Cell Type	Correlation	P
rs9891567	0.80	A/G	0.76	0.67–0.87	3.2*10^−5^	Multiple Tissue Human Expression Resource^4^	Lymphoblastoid cells	−0.58	7.7*10^−8^
							Adipocytes	−0.46	2.0*10^−5^
							Skin cells	−0.50	4.2*10^−6^
rs1533031	0.57	A/G	0.80	0.71–0.91	3.2*10^−4^	Geneva Umbilical Cord Bank^5^	Lymphoblastoid cells	−0.39	5.0*10^−4^
							T-cells	−0.36	1.6*10^−3^
							Fibroblasts	0.02	0.85

Abbreviations: r^2^: linkage disequilibrium r^2^ measure; OR: odds ratio; 95%CI: 95% confidence interval; P: p-value; Correlation: Pearson correlation coefficient.

1 Linkage disequilibrium r^2^ measure with rs6502976 in 250 unrelated African American controls from this study; SNP rs6502976 was not genotyped in either study.

2 Minor allele in African Americans bolded; modeled by generalized estimating equations adjusting for percent global West African ancestry and sex.

3 Pearson correlation values for genotype by *XAF1* expression level (Illumina probe identifier ILMN_2370573); the direction of the correlation corresponds to an increasing numbers of the minor allele in African Americans, which is the allele that is associated with sarcoidosis risk reduction.

4 Nica et al 2011. Correlation results reported for twin 1; results were consistent for twin 2.

5 Dimas et al 2009. SNP rs9891567 was not genotyped as part of this study.

### Immunohistochemistry (IHC) studies of *XAF1* and *XIAP*

**Figure 3:**
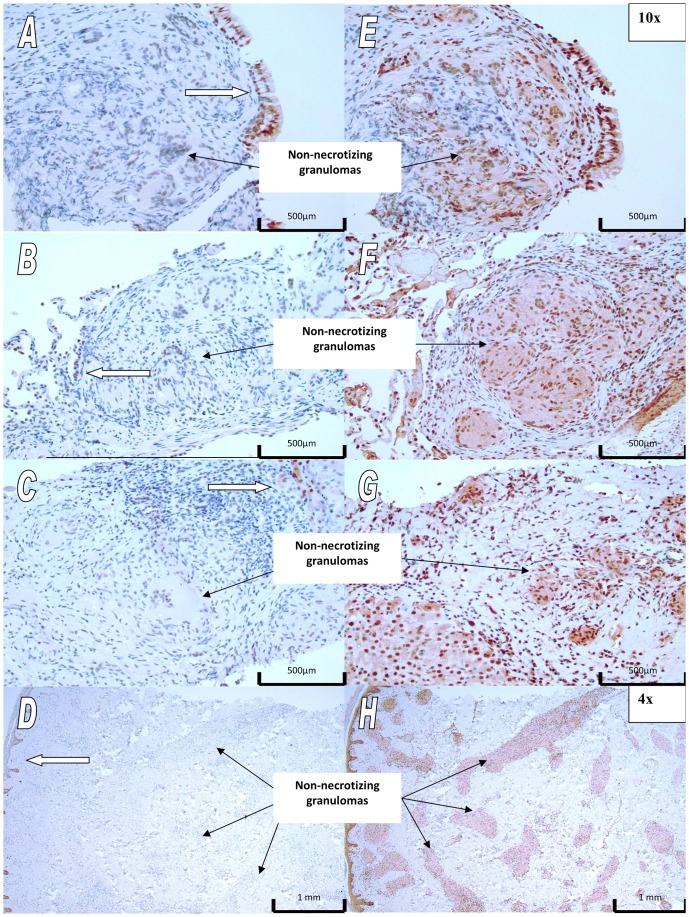
Representative pictures of XAF1 and XIAP staining of sarcoidosis-affected tissues. Panels A–D depict XAF-1 staining; panels E–H depict XIAP staining. Panels A and B are bronchial mucosa; E and F are lung tissue; C and G are liver tissue; and D and H are skin tissue. In general, XAF1 staining is negative in sarcoidosis-affected areas and limited to epithelial cells at the periphery (white arrows). XIAP staining was positive, with greater intensity observed in non-caseating granulomas.

To further explore *XAF1* as a novel sarcoidosis candidate susceptibility gene in African Americans, we conducted IHC protein expression studies for both the *XAF1* and X-linked inhibitor of apoptosis (*XIAP*) genes in granulomatous sarcoidosis-affected tissue. We stained thirteen sarcoidosis-affected tissue specimens (7 lung, 2 lymph nodes, 1 liver, 1 spleen, 2 skin) from twelve African American patients. Nuclear and cytosolic staining for XIAP was positive in all specimens whereas XAF1 staining was consistently negative or very weak. Representative IHC results for both XAF1 and XIAP are shown in Figure 3. XAF1 expression (Figure 3a–d) is present at the periphery of the specimen, in histologically normal cells distal to granulomas; increased XIAP staining (3e–f) clearly demarcates the sarcoidosis granulomas.

### Estimation of heritability of sarcoidosis risk due to local ancestry overall and by radiographic phenotypes

To quantify the contribution of local West African ancestry to sarcoidosis risk, we used the GCTA approach to estimate disease heritability due to local ancestry across autosomes [15], [16]. The count of zero, one, or two West African alleles was used in place of the actual genotype to compute the covariance between individuals and to estimate the heritability of sarcoidosis (overall and by radiographic phenotype) due to local ancestry (Table 4). For comparison, the GCTA estimates of the additive genetic effects due to common SNPs (>1% minor allele frequency) are also provided. We observed that 15% of the variation in sarcoidosis genetic risk is due to local ancestry, compared to a heritability of 26% due to the additive effects of common variation. Stratifying the sarcoidosis cases by radiographic phenotype increased the local ancestry heritability point estimate for resolving disease (23%). The local ancestry heritability for Scadding stage IV disease was also higher (26%) than that for stage I–III (18%) disease.

**Table 4:** Heritability of sarcoidosis risk attributable to difference in local ancestry overall and by radiographic phenotypes.

						Admixture Locus Removed^1^					
		All Autosomes				6p24.3–12.1		17p13.3–13.1		2p12–q12.1	
Case Sub-groups	N	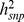 (SE)	P	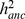 (SE)	P	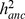 (SE)	P	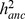 (SE)	P	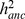 (SE)	P
Overall^2^	689	0.26(0.07)	3.1*10^−5^	0.15(0.04)	1.7*10^−4^	0.12 (0.05)	0.02	0.15 (0.04)	1.8*10^−4^	0.15(0.04)	1.8*10^−4^
Radiographic Phenotypes											
*Resolved*	226	0.53(0.15)	6.4*10^−5^	0.23(0.08)	0.018	0.15(0.11)	0.177	0.23(0.08)	0.018	0.22(0.08)	0.022
*All Persistent*	463	0.28(0.08)	3.6*10^−4^	0.17(0.04)	7.7*10^−4^	0.14(0.05)	0.019	0.17(0.04)	7.5*10^−4^	0.17(0.04)	7.1*10^−4^
* Scadding Stage I–III*	322	0.28(0.11)	0.003	0.18(0.06)	0.002	0.11(0.08)	0.137	0.19(0.06)	0.002	0.19(0.06)	0.002
* Scadding Stage IV*	141	0.73(0.26)	0.003	0.26 (0.13)	0.066	0.27(0.13)	0.059	0.25(0.13)	0.077	0.27(0.13)	0.063

Note: Number of controls (n = 859) is the same across case analysis strata.

Abbreviations: N: number of cases; 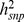: proportion of additive genetic variance due the common variants (minor allele frequency ≥1%); 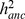:the proportion of the additive genetic variance due to local West African ancestry; SE: standard error of 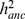; P: p-value from a one-degree-of-freedom likelihood ratio test of the additive genetic variance component.

1 For these analyses, the corresponding admixture locus was removed to estimate the effect on the heritability estimate.

2 These analyses were restricted to the subset of cases with a minimum of two years of follow-up.

To estimate the effects on heritability of the three admixture linkage regions (6p24.3–12.1, 17p13.3–13.1, and 2p12–q12.1) containing variants associated with sarcoidosis risk at or above the suggestive level of genome-wide significance (p = 10^−5^), the local ancestry estimates for these regions were each removed, and the heritability estimates were recalculated (Table 4). Unsurprisingly, the largest effect on the overall heritability estimate resulted from removal of the 6p24.3–12.1 region. Local ancestry over this region accounted for an approximately 20% reduction in the heritability estimate, indicating that ∼80% of the heritability to risk due to local ancestry is attributable genetic variation residing in areas of the genome outside of the broader major histocompatibility complex region. Removal of the other admixture-linked loci had less of an effect (∼0.2% reduction). For subgroups of radiographic phenotypes, removal of local ancestry at the 6p24.3–12.1 region resulted in lower heritability estimates for resolving (35% reduction) and persistent Scadding stage I–III (39% reduction) disease (Table 4); notably, however, removal of this region had little effect heritability estimate for persistent Scadding stage IV disease (Table 4).

## Discussion

Sarcoidosis incidence varies across populations of different ancestry, even within common geography, and is more common among people of West African ancestry. We have previously used admixture mapping to show that local West African ancestry is associated with disease risk in African Americans [12], [13]. In this study, we focused on previously-identified admixture regions, using genotyping data from our recently-published GWAS of sarcoidosis [14] and genotype imputation within the prioritized regions.

The SNP with the lowest p-value at the most significant novel admixture locus (17p13.3–13.1)—rs6502976—is located within intron 5 of the *XAF1* gene, a novel candidate risk gene for sarcoidosis. XAF1 is a negative regulator of XIAP, upregulating apoptosis by antagonizing the anti-caspase activity of XIAP [21]. XAF1 also antagonizes the cellular inhibitor of apoptosis genes *C-IAP1* and *C-IAP2* [22], and may sensitize cells to Fas-mediated apoptosis [23], which is thought to play a role in sarcoidosis [24], [25]. In IHC expression studies, we observed lack of XAF1 expression in sarcoidosis affected tissues and higher XIAP expression within sarcoid granulomas than in surrounding tissues. While we were unable to relate XAF1/XIAP expression levels to genotype, the staining patterns we observed suggest that inhibition of apoptosis as a result of low XAF1/high XIAP expression may influence granuloma formation or maintenance. Our analysis showed that rs6502976 was likely the only SNP responsible for the admixture linkage signal within this region. Further, eQTL analyses suggest a potential functional role for this SNP in the transcriptional expression of *XAF1*, which may affect XAF1 protein levels. Because XAF1 protein expression was low to absent in sarcoidosis-affected tissues, we hypothesize that any role rs6502976 plays in disease etiology would be early in pathogenesis, before sarcoidosis granulomas are histologically detectable.

Fine mapping within the *HLA* region identified an intronic *HLA-DRA* variant—rs74318745—as the most significant SNP within this region. In our GWAS, multiple genetically-identical (r^2^ = 1) SNPs were significantly associated with sarcoidosis risk [14], including a missense SNP (rs7192) in *HLA-DRA* that has been associated with risk of both rheumatoid arthritis and systemic lupus erythematosus [26], and a splice-acceptor variant (rs8084) associated with rheumatoid arthritis [27], [28]. Other genetically-identical SNPs include rs3129889, associated with multiple sclerosis [29], and rs2227139, associated with white blood cell count [30]. However, additional results suggest that SNP rs74318745 (or variants in high linkage disequilibrium) may not completely explain the admixture linkage signal within the region. This finding is consistent with our GWAS, which identified four additional independent variants associated with sarcoidosis risk within or near the genes *HLA-DQA1*, *HLA-DRB1*, and *NOTCH4* [14]. This scenario is similar to the initial identification of the prostate cancer admixture signal at 8q24 [31] and the subsequent identification of multiple independently-associated variants within this region of the genome via association mapping in additional ethnically diverse populations [32].

Among the three non-*HLA* admixture regions associated with risk of disease, the most significant SNP was located within the *MGAT4A* gene on chromosome 2. In a gene expression study of pulmonary sarcoidosis tissues and healthy lung specimens [33], MGAT4A was up-regulated 1.66-fold (p = 0.0145, uncorrected for multiple testing) in sarcoidosis tissue.

For the admixture regions associated with risk of Scadding stage IV disease, the most significant SNP (rs12919626) was located within the *FA2H* gene located at chromosome 16q23.1. This gene catalyzes a critical hydroxylation step necessary for the formation of 2-hydroxy fatty sphingolipids, believed to be involved in cell signaling [34]. Increased *FA2H* gene expression has been observed in injured lung tissue in rats [35], [36]. As our results suggested that more than one variant in the region was likely to explain the admixture signal, analyses conditional on SNP rs12919626 revealed two variants associated with Scadding stage IV disease within the *WWOX* gene, with one (rs1077963) being the most likely candidate to explain the admixture linkage in the region. A known tumor suppressor gene (42, 43), *WWOX* resides with the second most common fragile site in the human genome [37], [38]. This gene was also recently found to be associated with lung function in a GWA meta-analysis [39], and a functional copy number variant was associated with lung cancer risk in a Chinese population [40].

While the peak SNP (rs906233) association at the 10p12.1–11.21 Scadding stage IV admixture locus is located in an inter-genic region, the genes flanking it have plausible roles in sarcoidosis. The lysozyme-like 2 (*LYZL2*) gene is part of a family of lysome-like genes that are bacteriolytic and play a protective role in host defense [41]. Also, *MAP3K8* is a gene known to activate nuclear factor kappaB production, which is a master regulator of genes involved in immune response [42].

Our local ancestry-based GCTA heritability results suggest that variation in linkage disequilibrium with local West African ancestry explains a large proportion of the heritable component of sarcoidosis risk among African Americans. Further, even after removing the three risk-associated admixture loci, there remained a sizable statistically significant proportion of variation in heritable risk attributable to the remaining local ancestry. The heritability analysis also showed that differences in local ancestry were associated with persistent disease, especially persistent Scadding stage IV disease, which is more prevalent among African Americans. These findings suggest that significant differences exist in the genetic architecture of sarcoidosis risk between African Americans and European Americans. In particular, removal of the local ancestry effect at the *HLA* region did not change the heritability estimates for risk of Scadding stage IV disease; this suggests that the variants in *HLA* region that explain the admixture linkage peak reside in genes that affect disease susceptibility more than disease progression.

The current study is not without limitations, the most notable being the lack of validation for the association findings. While we have validated the imputed variants using direct genotyping, the variants associated with risk and Scadding stage IV disease will need to be validated in additional association studies of sarcoidosis in African Americans. Scadding staging was assessed with chest roentgenograms. Although computed tomography is more sensitive for detecting fibrotic changes in the lungs of sarcoidosis patients [43], the number of missed Stage IV cases is likely small [44]. Given the large number of Scadding Stage IV cases in our analysis (n = 190), such misclassification would likely have nominal effects on our results. Another limitation of the study is the lack of direct genotyping of novel variants in the full sample and our reliance on an imputation-based approach to fine map the selected admixture loci. While additional sequencing in these regions would be ideal, we believe we have identified the most likely variants underlying the admixture signals in these regions—which can be follow-up with targeted sequencing.

In summary, we offer initial evidence for several potential novel non-*HLA* genes associated with sarcoidosis susceptibility and severity in African Americans. Furthermore, our ancestry heritability results suggest there is still undiscovered genetic variation underlying disease risk linked with ancestry. Our results emphasize that admixture mapping of ancestry-associated risk loci can identify important risk variants that go undetected in GWAS. Variation at the most promising novel sarcoidosis susceptibility gene, *XAF1*, may explain in part why African Americans are at increased risk for sarcoidosis. Validation studies of our *XAF1* association in independent samples as well as additional XAF1 functional studies are needed to further validate and define the role of this novel gene in sarcoidosis pathogenesis.

## Materials and Methods

### Ethics Statement

Table 5 describes our sample comprising 2,727 self-identified African Americans (1,271 cases, 1,456 controls) from three independent studies of sarcoidosis patients, family members, and controls: 1) a case-control etiologic study of sarcoidosis (ACCESS) [45]; 2) a multi-site affected-sibling pair sarcoidosis linkage study [46]; 3) a nuclear family-based sample ascertained through a single affected individual within the Henry Ford Health System in Detroit, MI [47]; and 4) healthy controls from the Oklahoma Medical Research Foundation (OMRF) Lupus Family Registry and Repository in Oklahoma City, OK [48]. For each of these studies, participants gave written informed consent to allow their research material to be used in future genetic studies. Study protocols were approved by the institutional review board of each study site (Beth Israel Deaconess Medical Center, Boston, MA; Cleveland Clinic, Cleveland, OH; Emory Healthcare, Atlanta, GA; Georgetown University Medical System, Washington, DC; HFHS, Detroit, MI; Johns Hopkins Hospital, Baltimore, MD; Medical University of South Carolina, Charleston, SC; Mount Sinai Hospital, New York, NY; National Jewish Hospital, Denver, CO; University of Cincinnati Hospital, Cincinnati, OH; University of Iowa Health Care, Iowa City, IA; University of North Carolina Medical Center, Chapel Hill, NC; University of Pennsylvania Health System, Philadelphia, PA;). DNA specimens were processed at OMRF.

**Table 5:** Demographic and clinical characteristics of the study sample.

Characteristic	Affected (n = 1,271)	Unaffected (n = 1,456)
Male n(%)	322 (25.3)	400 (27.5)
Percent African ancestry^1^	82.7 (9.4)	82.3 (11.1)
Radiographic phenotype^2^		
*Resolved*	308 (33.1%)	-
*All persistent*	623 (66.9%)	-
* Stage I–III*	433 (69.5%)	-
* Stage IV*	190 (30.5%)	-

1 Mean (standard deviation).

2 Two-year follow-up chest x-ray and Scadding stage data n = 931.

### Study Sample Ascertainment and Phenotyping

Sample ascertainment protocols and demographics have been described previously [45], [46], [47]. Where possible, cases were phenotyped as to the persistence or absence of radiographic evidence for lung disease two years after date of diagnosis. The procurement of these data was done retrospectively, except for cases enrolled during the first two years of the ACCESS study, when study protocol dictated a two-year follow-up exam [49]. For cases presenting with Scadding stage IV chest radiographs (evidence of lung fibrosis or scarring), no follow-up chest x-ray was needed for phenotyping (as stage IV x-ray indicates permanent changes). Follow-up data were missing on 26.8% of cases (340/1,271) due to the lack of necessary observation time between diagnosis and study enrollment (n = 196) or missing chest x-ray data at two or more after diagnosis (n = 144).

### Genotyping and imputation methods

Genotyping was performed at OMRF using the Illumina (San Diego, CA) HumanOmni1 Quad array for ∼1.1 M SNPs as part of our prior genome-wide association study [14]; details of genotyping and quality control have been previously described. Briefly included SNPs met the following quality control criteria: well-defined cluster plots by visual inspections; call rate >95%; minor allele frequency >0.01; Hardy-Weinberg proportion tests *P*>0.0001 in cases and *P*>0.001 in controls; and differences in case-control missingness *P*>0.001. Samples were removed from analysis for the following: duplicate of another sample; cryptic relatedness in independent datasets (proportion of alleles identical by descent >0.25); low call rates (<90%); extreme heterozygosity (>5 standard deviations from the mean); outlying principal component values of population membership (calculated by EIGENSOFT 3.0) [50] or global ancestry estimates (calculated by ADMIXMAP [51], [52]); discrepancy between reported sex and genetic data.

Imputation was performed in 5 Mb bins across the genome using the IMPUTE2 program [53] with 1000 Genomes Project Phase I data (release June 2011) [54]—which contains haplotypes derived from 1,094 individuals from Africa, Asia, Europe, and the Americas—as the reference. IMPUTE2 was used to estimate the posterior probabilities for the three possible genotypes (i.e. AA, AB, and BB); a threshold of 0.9 was applied to these posterior probabilities to produce the most likely genotypes. Imputed SNPs with low imputation accuracy (information measure <0.5 and average maximum posterior genotype call probability <0.9) or failing the above quality control standards were removed to minimize false positives.

We used imputation data for the four regions previously associated with sarcoidosis risk (2p12–q12.1, 6p24.3–12.1, 6q23.3–25.2, and 17p13.3–13.1) and three regions associated with Scadding stage IV disease (2p12–q12.3, 10p12.1–11.21, and 16q21–23.2). Table S2 displays the variants analyzed in each region by genotype/imputation status. For imputed variants, we include a summary of the imputations which exceeded a quality threshold of 0.9; if the primary SNP in a region was imputed, we confirmed accuracy through direct genotyping in a sub-sample of individuals. There were four such SNPs. One (rs6502976) was confirmed in a sub-sample of 426 individuals via sequencing, using the Illumina (San Diego, CA) HiSeq2000 platform with Illumina Pipeline software (version 1.7). The remaining three SNPs (rs62158012, rs6547087, and rs12919626) were confirmed in a sub-sample of 475 individuals using the TaqMan (Applied Biosystems; Foster City, CA) allelic discrimination technology. The agreement results (overall and by genotype) are presented in Table S3 and indicated strong overall agreement with imputation (≥98%) for all four SNPs. In the text, we also report the association result for the genotyped SNP in highest pairwise LD (as measured by r^2^) with the primary imputed SNP, where r^2^ was calculated on a sub-sample of 250 unrelated African American controls from this study.

### Statistical Analysis

Our original admixture scan in a family-based sample identified a total of twelve regions of interest: nine associated with risk of disease and three associated with Scadding stage IV disease [12]. While this original analysis required selection of a single affected individual from a family, for our analysis we used a new application of ADMIXMAP that permits inclusion of all affected family members to maximize statistical power [55]. Based on these most recent admixture mapping results, fine-mapping was restricted to those regions for which we could not exclude an ancestry risk ratio of ≥2 or ≤0.5 (at a base-10 logarithm of the odds admixture linkage score of -2, based on Hoggart et al's exclusion-mapping approach) [52]. This resulted in four regions associated with sarcoidosis risk (2p12–q12.1: 71,618,323–106,550,301; 6p24.3–12.1: 18,069,307–44,536,360; 6q23.3–25.2: 134,423,766–144,455,085; 17p13.3–13.1: 0–11,993,789) and three regions associated with Scadding stage IV disease (2p12–q12.3: 80,127,798–112,062,746; 10p12.1–11.21: 24,687,265–35,999,931; 16q21–23.2: 65,774,387–79,031,043). The listed base-pair region boundaries for association testing were determined by the first and last marker with affected-only admixture p-values <0.05.

The Local ancestry in AdMixed Populations (LAMP) method [56], [57] was used to estimate local ancestry—defined as the probability of carrying zero, one, or two copies of west African (or European) ancestral alleles at each SNP across the genome for each individual; this method implements a sliding-window approach, using allele frequencies of genome-wide markers in the underlying ancestral populations to guide the estimation. Estimates of ancestral allele frequencies for Illumina Omni-Quad SNPs were derived from the HapMap [58] Yoruba and CEPH European Utah catalogs, available through the Illumina iControl database. The LAMP linkage disequilibrium threshold value for this analysis was r^2^ = 0.1. Each window of local ancestry estimation overlapped 20% of the markers in the adjacent windows, and a constant recombination rate of 10^−8^ per base pair was assumed. Imputation of local ancestry for markers between non-linkage disequilibrium-filtered markers was based on majority vote from the local ancestry estimates of overlapping windows. For SNPs imputed using the haplotypes from the 1000 Genomes Project catalog and not included in the GWA genotyping, imputation of local ancestry was based on the nearest genotyped SNP, with local ancestry estimated via LAMP.

To use the complete sample of related and unrelated individuals for association fine-mapping within regions of confirmed admixture linkage, generalized estimating equations with logit link function and an independence working correlation matrix were used to compute the odds ratio for each SNP under a multiplicative model (i.e. log additive), treating each family as a cluster [59]. Because the local ancestry association signal may confound these estimates, odds ratios were computed both with and without adjustment for local ancestry; the degree of confounding was calculated as the absolute difference between adjusted and unadjusted log odds ratios, divided by the unadjusted log odds ratio. Additionally, covariates for genome-wide West African ancestry and sex were included in all models.

Next, markers with p-values <0.05 that displayed minimal confounding by local ancestry were tested using the MIX score approach [11]. The MIX score tests the likelihood that a given SNP explains an ancestry signal by constructing a test of the ancestry odds ratio, parameterized by the allelic odds ratio conditional on local ancestry and the underlying ancestral allele frequencies. The null distribution of the MIX score is a one degree of freedom chi-square and assumes that a single causal explains the admixture linkage in a region. The degree to which this assumption is met may be tested by a one degree of freedom difference score (DIFF) between the MIX score and the sum of the independent affected-only admixture score and the allelic SNP association score, conditional on local ancestry signal; therefore, a DIFF score p-value less than 0.05 indicates that there is likely more than one SNP responsible for the local ancestry signal. Because the MIX score assumes cases and control are unrelated, we performed one hundred random, independent samples of 1,779 unrelated subjects (933 cases, 846 controls); the SNP-specific MIX score statistic was calculated as the average of these 100 samples.

This tiered analytical approach (i.e. refinement of region of the genome where association testing is carried out based on affected-only admixture mapping results) takes advantage of the independence between the local ancestry and the marker genotype associations conditional upon local ancestry, resulting in testing many fewer marker genotype associations than in a traditional genome-wide association study. Therefore, we emphasize only the results of those variants that met the established genome-wide significance threshold of 5*10^−8^, the suggestive threshold of 10^−5^, and/or those most likely to explain the admixture linkage within each region.

Additionally, we used the Genome-wide Complex Trait Analysis (GCTA) program [15], [16] to calculate a genome-wide ancestry-based relationship matrix and to estimate from the proportion of variance in liability to sarcoidosis that is explained by additive effects of local ancestry. The same argument used by Yang et al. [60] to estimate the genetic variance attributable to SNPs can be used to estimate the genetic variance attributable to local ancestry. For comparison, we also estimated the variance attributable to genotyped autosomal SNPs. For both analyses, a sarcoidosis prevalence of 1/1000 was used. To exclude the effects of shared environment and alleles shared within families, the dataset was restricted to individuals whose coefficient of relationship was calculated from the pedigree to be less than 0.125 (equivalent to first cousins) using a method described in Manichaikul et al [61] and implemented in the KING relationship inference software [62]. The analyses controlled for genome-wide ancestry proportion and sex. Because African Americans are more likely to have persistent sarcoidosis than Europeans Americans [7], [8], [63], we also investigated whether radiographic phenotypes (resolution of disease after a minimum of two years of follow-up; persistence of disease after this time with Scadding stage IV disease; persistent disease without Scadding stage IV; Scadding stage IV disease alone) differed in heritability associated with local ancestry differences. In this analysis, each category was compared to controls.

### Immunohistochemistry

Specimens of lung, liver, spleen, lymph node, and skin tissue from twelve African American patients with histologically-confirmed sarcoidosis were procured from the HFHS Department of Pathology. Each specimen was mounted on a slide, hemotoxin and eosin stained, and examined by the study pathologist (DAC) for presence of non-caseating granulomas. Rabbit polyclonal anti-XAF1 antibody (ProSci Incorporated, Poway, CA, USA) was diluted to 1∶300. Goat polyclonal anti-XIAP antibody (R & D Systems, Minneapolis, MN, USA) was diluted 1∶100. Immunohistochemical staining was performed using a standard avidin–biotin complex method with a streptavidin–biotin–peroxidase kit (Nichirei, Tokyo, Japan). Diaminobenzidine was used as a chromogen.

## Supporting Information

Figure S1 Geneva Umbilical Cord Bank* eQTL results for SNP rs1533031 and XAF1. Using the Genvar analysis tool, expression levels of XAF1 (Illumina probe identifier ILMN_2370573) are plotted by SNP rs1533031 genotype for each individual (n = 75) by cell type in umbilical cord samples. Abbreviations: r, Pearson correlation coefficient; P. *Dimas et al 2009.
(EPS)

Figure S2 Multiple Tissue Human Expression Resource* eQTL results for SNP rs9891567 and XAF1. Using the Genvar analysis tool, expression levels of XAF1 (Illumina probe identifier ILMN_2370573) are plotted by SNP rs9891567 genotype for each identical twin (n = 171 female identical twins) by tissue type. Abbreviations: r, Pearson correlation coefficient; P. * Nica et al 2011.
(EPS)

Table S1 **Association results for markers with local ancestry-adjusted or –unadjusted p-values <0.05.** Case-control association results are shown for the following loci: Chr 2q11.2, Chr 6p21.32, Chr 6q23.3 and Chr 17p13.1. Stage IV case association results are shown for the following loci: Chr 2p12, Chr 10p11.23 and Chr 16q23.1.
(XLS)

Table S2 **Number of variants analyzed by admixture locus and imputation status.**
(DOCX)

Table S3 **Confirmation genotyping of imputed SNPs rs62158012 (Chr 2p12–q12.1), rs6502976 (Chr 17p13.3–13.1), rs6547087 (Chr 2p12–q12.3) and rs12919626 (Chr 16q21–23.2).**
(DOCX)
