# Supplementary material for: Admixture Fine-Mapping in African Americans Implicates XAF1 as a Possible Sarcoidosis Risk Gene
Source: PLoS One. 2014 Mar 24;9(3):e92646. doi: 10.1371/journal.pone.0092646 (PMC3963923; doi:10.1371/journal.pone.0092646)
Supplement: Table S2 — Number of variants analyzed by admixture locus and imputation status. (DOCX) [file pone.0092646.s004.docx]

**Supplementary Table 2: Number of variants analyzed by admixture locus and imputation status.**

|  |  |  |  | Genotyped | | Imputed | |
| --- | --- | --- | --- | --- | --- | --- | --- |
|  |  |  |  |  |  | All | Information >0.9 |
| Outcome | Locus | Base-Pair Region* |  | Total | N (%) | N (%) | N (%†) |
|  |  |  |  |  |  |  |  |
| Risk | 2p12–q12.1 | 71,618,323–106,550,301 |  | 162,205 | 7,713 (4.8%) | 154,492 (95.2%) | 104,003 (65.5%) |
|  | 6p24.3-12.1 | 18,069,307–44,536,360 |  | 178,943 | 21,122 (11.8%) | 157,821 (88.2%) | 117,482 (74.4%) |
|  | 6q23.3–25.2 | 134,423,766–144,455,085 |  | 50,137 | 4,679 (9.3%) | 45,458 (90.7%) | 34,640 (76.2%) |
|  | 17p13.3–13.1 | 0–11,993,789 |  | 40,792 | 4,679 (11.5%) | 36,113 (88.5%) | 29,222 (80.9%) |
| Scadding Stage IV | 2p12–q12.3 | 80,127,798–112,062746 |  | 142,933 | 6,237 (4.4%) | 136,696 (95.6%) | 87,755 (61.4%) |
|  | 10p12.1–11.21 | 24,687,265–35,999,931 |  | 68,016 | 3,653 (5.4%) | 64,363 (94.6%) | 49,995 (77.7%) |
|  | 16q21–23.2 | 65,774,387–79,031,043 |  | 75,807 | 4,120 (5.4%) | 71,687 (94.6%) | 51,455 (71.8%) |
|  |  |  |  |  |  |  |  |

*Human Genome Build 19.

† Percent of the total number of imputed variants.
