# Supplementary material for: Admixture Fine-Mapping in African Americans Implicates XAF1 as a Possible Sarcoidosis Risk Gene
Source: PLoS One. 2014 Mar 24;9(3):e92646. doi: 10.1371/journal.pone.0092646 (PMC3963923; doi:10.1371/journal.pone.0092646)
Supplement: Table S3 — Confirmation genotyping of imputed SNPs rs62158012 (Chr 2p12–q12.1), rs6502976 (Chr 17p13.3–13.1), rs6547087 (Chr 2p12–q12.3) and rs12919626 (Chr 16q21–23.2). (DOCX) [file pone.0092646.s005.docx]

**Supplementary Table 3: Confirmation genotyping of imputed SNPs.**

|  |  |  |  |  |  | Count (% Agreement With Genotyping) | | | | |
| --- | --- | --- | --- | --- | --- | --- | --- | --- | --- | --- |
| Outcome | Locus | SNP* |  | Alleles* | Imputation  Information† | AA | AB | BB |  | Total‡ |
|  |  |  |  |  |  |  |  |  |  |  |
| Risk | 2p12–q12.1 | rs62158012 |  | **A**/C | 0.97 | 390 (99.2%) | 63 (98.4%) | 9 (100.0%) |  | 462 (99.1%) |
|  | 17p13.3–13.1 | rs6502976 |  | **C**/G | 0.98 | 235 (99.2%) | 141 (97.2%) | 20 (91.0%) |  | 396 (98.0%) |
|  |  |  |  |  |  |  |  |  |  |  |
| Scadding Stage IV | 2p12–q12.3 | rs6547087 |  | **C**/T | 0.99 | 374 (98.9%) | 68 (100.0%) | 11 (100.0%) |  | 453 (99.1.0%) |
|  | 16q21–23.2 | rs12919626 |  | **A**/G | 0.96 | 365 (99.5%) | 73 (100.0%) | 15 (100.0%) |  | 453(99.6%) |
|  |  |  |  |  |  |  |  |  |  |  |

Abbreviations: AA: major allele homozygote; AB: heterozygote; and BB: minor allele homozygotes.

*Minor allele in African Americans is bolded.

†IMPUTE2 imputation information.

‡Total count not equal to 475 (rs62158012, rs6547087, and rs12919626) or 426 (rs6502976) occurred due to missing genotype data from imputation and/or direct genotyping.
